# Use of Specific Chemical Reagents for Detection of Modified Nucleotides in RNA

**DOI:** 10.4061/2011/408053

**Published:** 2011-04-13

**Authors:** Isabelle Behm-Ansmant, Mark Helm, Yuri Motorin

**Affiliations:** ^1^Laboratoire ARN-RNP Maturation-Structure-Fonction, Enzymologie Moléculaire et Structurale (AREMS), UMR 7214 CNRS-UHP, Nancy Université, boulevard des Aiguillettes, BP 70239, 54506 Vandoeuvre-les-Nancy, France; ^2^Institute of Pharmacy and Biochemistry, Johannes Gutenberg-University of Mainz, Staudingerweg 5, 55099 Mainz, Germany

## Abstract

Naturally occurring cellular RNAs contain an impressive number of chemically distinct modified residues which appear posttranscriptionally, as a result of specific action of the corresponding RNA modification enzymes. Over 100 different chemical modifications have been identified and characterized up to now. Identification of the chemical nature and exact position of these modifications is typically based on 2D-TLC analysis of nucleotide digests, on HPLC coupled with mass spectrometry, or on the use of primer extension by reverse transcriptase. However, many modified nucleotides are silent in reverse transcription, since the presence of additional chemical groups frequently does not change base-pairing properties. In this paper, we give a summary of various chemical approaches exploiting the specific reactivity of modified nucleotides in RNA for their detection.

## 1. Introduction

Native cellular RNAs contain numerous modified residues resulting from specific action of various RNA modification enzymes. These RNA modifications are ubiquitous in nature, but the specific modification profile varies depending on the organism. Over 100 chemically distinct modified nucleotides have been identified so far mostly in tRNAs, rRNAs, snRNAs and some snoRNAs. From the chemical point of view, these modifications are highly diverse and almost any position of the nucleobases as well as the 2′-OH of the ribose has been found to be a target of modification enzymes (see Table 1 and below) [1–3]. 

Identification of the chemical nature and localization of the modified nucleotides even in highly abundant RNAs represents a laborious and time-consuming task. Moreover, the analysis of low abundant cellular RNAs is extremely difficult due to limited access to highly purified RNA species required for most types of analysis, like HPLC or mass spectrometry [4–7]. One alternative to this consists in direct analysis of underrepresented RNA species in total cellular RNA by reverse transcription (RT) using specific DNA primers [8]. This generally allows the sequencing of a given RNA, but the information on its modified nucleotide content is still missing. The use of specific chemical reagents reviewed in this survey explores the particular reactivity of a given modified residue and may considerably help in the interpretation of an RT profile. Another area for the use of specific chemical reactions is RNA analysis and sequencing by various types of mass spectrometry (MS), where modified residues undergo particular fragmentation pathways and their derivatization helps in identification.

## 2. General Reactivity of Unmodified Nucleotides

Detection of nucleotide modifications is based on either of three basic principles. For two of these, that is, separation according to physicochemical properties and differential enzymatic turnover, we refer to other reviews [4, 7, 9–12] and will mention such methods only when they are combined with the use of chemicals that specifically react with modified nucleotides. Here, we focus on the third principle, which is differential chemical reactivity. To set the stage, we will outline the known reactivity of the four major ribonucleotides in this paragraph. In principle, any reagent that reacts with nucleotides may be considered for chemical recognition of nucleotide modification, provided that conditions can be determined, where its reactivity significantly differs between a given major nucleotide and its modified counterpart. In the best case, conditions would be optimized to the point of exclusive reaction with either the standard or the modification. From a practical point of view, it is helpful to distinguish reagents within a narrowly defined window of experimental conditions from those which reliably and completely discriminate nucleotides over a wide plateau of experimental conditions. The former methods are evidently more difficult to newly establish in a laboratory, despite the emergence of numerous publications covering detailed protocols, and should by tackled only by experienced RNA scientists. However, even for the less complicated reactions, one should be conscious that deviations in temperature, pH, salt, incubation time, or reagent concentration may result in “leaving” the plateau and thus result in suboptimal discrimination. Similar considerations apply to the detection of the chemical species resulting for treatment with the discriminating reagent. Most often, the ease and turnaround time of detection will determine if a given reagent finds widespread use in the RNA community. 

The above considerations apply to reagents of various types alike, which we have grouped into electrophiles, nucleophiles, and oxidizing, and reducing agents. 

### 2.1. Electrophilic Reagents

**Figure 1:**
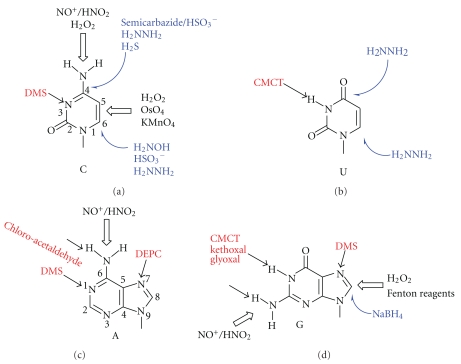
General chemical reactivity of nucleobases. Attack sites on the nucleobase are indicated by arrows. Open black arrows indicate oxidizing agents, filled black ones indicate alkylating electrophiles, and blue ones indicate nucleophiles. Numbering of the nucleobase atoms 1–6 for pyrimidines or 1–9 for purines is indicated outside the ring.

Nitrogen atoms in purine and pyrimidine rings of nucleobases show varying degrees of nucleophilicity and thus react differentially with various electrophilic compounds, which are typically alkylating or acylating agents (Figure 1). 

These reactivities have been explored decades ago and form the basis of chemical sequencing of end-labeled nucleic acids according to Maxam and Gilbert [13–15]. Methylating agents, in particular dimethylsulfate (DMS), preferentially alkylate the *N1* of adenosines, the *N7* of guanosines, and the *N3* of cytidines. All three sites are also found to be enzymatically methylated *in vivo*, and the methods used for sequencing of chemical probing of RNA structure can also be applied to reveal these naturally occurring modifications. An important property of many modified nucleotides is that they render oligonucleotides susceptible to chain scission via *β*-elimination, provided that the modification itself or an auxiliary chemical treatment ablates aromaticity of the base or leads to abasic sites. *β*-elimination is induced by heating of RNA with aniline at low pH. Additional treatment of m^3^C with hydrazine and of m^7^G [16], dihydrouridine, and wybutosine with sodium borohydride (NaBH_4_) leads to abasic sites in RNA which are susceptible to aniline cleavage. The wybutosine base is also acid-labile and will undergo depurination upon treatment with hydrochloric acid to leave an abasic site [17]. To date, there is no satisfactory protocol to chemically reveal the presence of *N1*-methyladenosine in RNA, which therefore has to be detected by RT-based methods, as will be detailed below. 

Other popular electrophilic agents that react with nitrogens include diethyl pyrocarbonate (DEPC, *N7* of adenosine) and ethylnitrosourea, which alkylates all nitrogens in addition to phosphates and all oxygens in nucleic acids [18]. *N*,*N*-(dimethylamino) dimethylchlorosilane (DMAS-Cl) reacts with the *N^2^* of guanosines [19]. Certain reagents gain in specificity due to the presence of two electrophilic groups, for example, chloroacetaldehyde (*N1*-A,* N^6^*-A *N3*-C,* N^4^*-C) [20, 21], chlorotetrolic (4-chloro-2-butynoic) acid ester (*N1*-A,* N^6^*-A *N3*-C,* N^4^-*C) [22], and glyoxal and kethoxal (*N1*-G, *N^2^*-G) [23, 24]. Finally, carbodiimides, in particular 1-cyclohexyl-3-(2-(4-morpholinyl)ethyl) carbodiimide tosylate (CMCT), acylate the *N3* of uridines and the *N1* and *N^2^* of guanosines, as well as both nitrogens in pseudouridine and *N3* in inosine. Its use for the mapping of Ψ residues will be detailed below.

### 2.2. Oxidizing Agents

As electron poor species, oxidizing agents share certain properties and also target moieties with alkylating agents. Nitrosyl cations and related species react with exocyclic nitrogens in cytidine, adenosine and guanosine to form diazonium compounds, which eventually hydrolyze and yield the corresponding deamination products: uridine, inosine, and xanthene, respectively. Monoperphtalic acid was reported to oxidize adenosine to yield adenosine 1-*N*-oxide [25]. The Δ5,6 double bonds in pyrimidines can in principle be oxidized by hydrogen peroxide although permanganate and in particular osmium-VIII compounds are most frequently used (reviewed in [26]).

Radical generating species, including Fenton reagents [27], copper phenantroline [28, 29] and peroxonitrous acid [30] are known to abstract hydrogens from CH bonds in the ribose. So far, this is the only type of reactivity that has not been exploited for the detection of nucleotide modifications. This type of reagents also causes the formation of 8-oxo-guanosine, which is the major lesion in oxidative damage of DNA [31].

### 2.3. Nucleophilic and Reducing Reagents

In pyrimidines, the Δ5,6 double bond is part of a Michael acceptor, which can be attacked in the 6 position by strong nucleophiles such as, bisulfite, hydrazine, methoxamine, hydroxylamine [32–34], and bisulfite [35–37] but also by KI/TiCl_3_ [38]. NaBH_4_ will act only on methylated *N3*-C, as pointed out already. 

The C4 position is also electrophilic, but typically less reactive than the Michael acceptor electrophile C6. Nucleophilic attack here is known from the bidentate hydrazine [39], or a combination of bisulfate and a semicarbazide [40]. In both cases, the first nucleophilic attack occurs at the C6, and only then does the C4 position react [33, 39]. Using hydrogen sulfide under high pressure, cytidines can be converted into s^4^U as the result of a nucleophilic attack at the carbon 4 position [41, 42], which might be preceded by an initial nucleophilic attack at the C6.

## 3. Specific Reactivity of Modified Nucleotides

**Figure 2:**
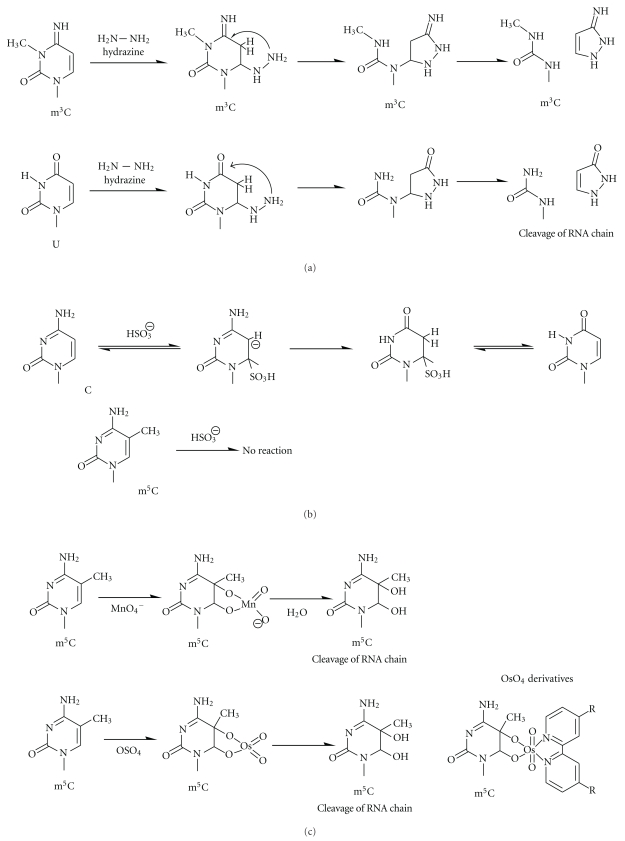
Specific chemical reactions for m^3^C and m^5^C detection. (a) Cleavage of m^3^C and unmodified uridine by hydrazine. (b) Deamination of unmodified cytosine by bisulfate; m^5^C is resistant for deamination. (c) Specific oxydation of 5-6 double C=C bond in methylated pyrimidines by MnO_4_^−^, OsO_4_, and OsO_4_ derivatives.

From a perspective of chemical reactivity, it is helpful to distinguish between modifications carrying principally new chemical moieties, and those which only moderately alter the reactivity of chemical moieties already present in RNA. The latter category essentially contains all methyl group additions, and reagents and reaction conditions must be carefully controlled to achieve discrimination. Thus, the detection of m^3^C, which has already been mentioned (see Figure 2(a)), is based on the susceptibility of cytidines towards nucleophiles, which was increased by the methyl group modification to *N3*, and thus allowed for detection after hydrazine treatment. In contrast, addition to the 5 position of a methy lgroup renders cytidines more electron rich and consequently less electrophilic. Therefore, nucleophilic attacks by bisulfite can be restricted to cytidines under carefully controlled conditions which leave m^5^C unaffected by bisulfite.

A number of more sophisticated modifications involve the introduction of chemical moieties which are chemically orthogonal, that is, they can be conjugated by certain reagents to which unmodified RNA is completely inert. Thus, isothiocyanate and NHS-derivatives selectively react with primary amines as present, for example, in acp^3^U, or lysidine (k^2^C). Thiolated nucleotides such as s^2^U, s^4^U, and s^2^C [43, 44] react with iodoacetamide derivatives, and carboxylic acids such as in t^6^A/m^6^t^6^A and acp^3^U [45, 46], other COOH-containing nucleotides can most probably be activated by carbodiimides for conjugation with a nitrogen nucleophile. 

### 3.1. 5-Methylcytosine

The chemistry of m^5^C in DNA has been subject to intense research as a consequence of the importance of m^5^C in the field of epigenetics. Bisulfite m^5^C sequencing is a state-of-the art detection method and used on a very large scale. The addition of bisulfite to the 6 position promotes a series of chemical transformations depicted in Figure 2(b), which ultimately leads to deamination. Cytidines are thus transformed into uridines and read as such in subsequent sequencing reactions. Based on their resistance to deamination reaction, sequencing after bisulfite treatment will reveal m^5^C residues as cytidine signals. Despite lasting efforts, adaptation of bisulfite to RNA has only recently succeeded [35–37] and is now one of the few methods that might be used to investigate RNA modification on a genome-wide scale [47, 48].

As indicated in Figure 2(c), another avenue of chemical discrimination between cytidines and m^5^C may involve oxidation of the double bond. Although the oxidation with permanganate produces insoluble MnO_2_, and further oxidation of the glycol to bis-aldehyde may occur under certain conditions, it has been successfully employed in the discrimination of deoxy-m^5^C versus deoxycytidine [49, 50]. The osmium tetroxide reactions do not involve the formation of insoluble products, but the reagent is highly toxic and mutagenic. In the past years, a variety of osmate-based bioconjugate reagents have been developed for the selective detection of m^5^C in DNA. These reagents exploit the additional electron density brought about by the methyl group for highly selective formation of a stable complex with deoxy-m^5^C but not with deoxycytidine. However, applications to RNA are still lacking [51–53] (reviewed in [26]).

### 3.2. 3-Methylcytosine

Detection of m^3^C is based on its increased reactivity towards hydrazine, as discussed above. Figure 2(a) shows the reaction sequence leading to an intermediate susceptible to aniline-induced cleavage *via* *β*-elimination. Cleavage sites can be revealed by sequencing reactions with end-labeled RNA [14].

### 3.3. 7-Methylguanosine

**Figure 3:**
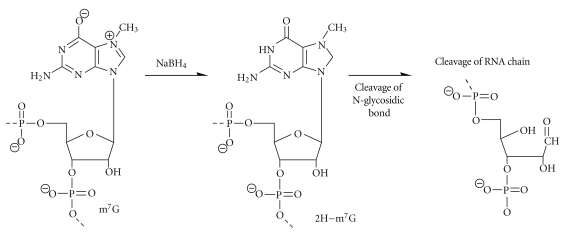
Sodium borohydride (NaBH_4_) reduction of m^7^G in RNA. Reduction of m^7^G leads to formation of abasic site in RNA followed by the cleavage of the RNA chain by *β*-elimination.

Guanosine methylated at position 7 (m^7^G) is frequently present in the variable region of tRNAs. As has been pointed out, its detection can be achieved by aniline-induced cleavage of the RNA chain by *β*-elimination after additional treatment under alkaline conditions [16] or after its reduction by sodium borohydride (NaBH_4_, shown in Figure 3). Because solutions of NaBH_4_ are typically alkaline, and because the reduction product has been described as sensitive to reoxidation in air, the actual species undergoing beta elimination may not be well defined. This reaction was first studied for isolation of defined tRNA fragments [54] and later applied to RNA sequencing using DMS in Maxam and Gilbert type chemistry [14] or RNA structural probing.

### 3.4. 2′-O-Methylated Riboses

**Figure 4:**
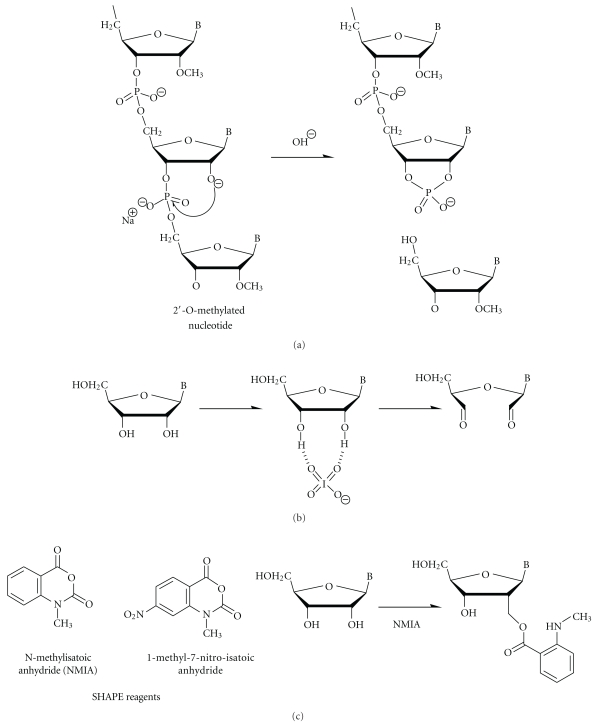
Specific reagents for 2′-*O*-methylated residues in RNA. (a) Selective cleavage of nonmethylated ribose residues at alkaline pH. The 2′-*O*-methylated residues are resistant for such cleavage. (b) Periodate oxydation of cisdiol residues at the terminal 3′-ribose. (c) Acylation of ribose free 2′-OH by SHAPE reagents N-methylisatoic anhydride (NMIA) and 1-methyl-7-nitro-isatoic anhydride.

Alkaline hydrolysis of RNA polynucleotide chains proceeds via deprotonation of the ribose 2′-OH to the corresponding alcoholate, and its nucleophilic attack of the nearby 3′-phosphate. The resulting intermediate is unstable and rapidly decomposes into a 2',3'-cyclophosphate leading to phosphodiester bond cleavage (Figure 4(a)). The methylation of ribose 2′-OH prevents alcoholate formation, and thus decreases the reactivity of the 2′-oxygen and consequently prevents phosphodiester bond cleavage almost completely [55, 56]. This resistance to alkaline hydrolysis of the phosphodiester bonds on the 3′-side of 2′-*O*-methylated nucleotides can be detected by primer extension or by direct analysis of the cleavage profile of end-labeled RNA. The 2′-*O*-methylated residue appears as a “gap” in the regular ladder of OH^−^ cleavage. 

A very sensitive method involving RNase H digestion directed by 2′-*O*-methyl RNA-DNA chimeras can be used to confirm the presence of a 2′-*O*-methylated residue. RNase H nonspecifically cleaves the RNA strand of an RNA-DNA hybrid. However, it does not cleave any site where the 2′-*O* position of an RNA residue is methylated. The digestion of an RNA directed by a complementary 2′-*O*-methyl RNA-DNA chimeric oligonucleotide consisting of four deoxynucleotides flanked by 2′-*O*-methyl ribonucleotides is site specific. The use of a 2′-*O*-methyl RNA-DNA chimera and RNAse H digestion therefore provide a direct assay for determining whether any particular nucleotide in a long RNA molecule carries a 2′-*O*-methyl group. If the 2′-*O* position of the targeted residue is methylated, RNAse H cleavage is blocked. If instead the 2′-*O* position is unmodified, RNAse H should cut the RNA specifically at that site [57].

Another method based on the RNA treatment by sodium periodate followed by *β*-elimination has been recently used to analyze the methylation status of the 3′-terminal nucleotide of plant miRNAs [58, 59]. The reactions eliminate a 3′ nucleotide containing both 2′- and 3′-OH groups on the ribose (Figure 4(b)), resulting in an RNA product that is one nucleotide shorter than the substrate RNA and that contains a 3′ phosphate group [60]. Therefore, miRNAs that contain a 3′ terminal ribose with both 2′- and 3′-OH groups will migrate faster during electrophoresis between one and two nucleotides after the treatment. Methylated miRNAs whose 3′ terminal ribose contains a 2′-*O*-methyl group do not participate in the reactions and remain unchanged in mobility. A significant disadvantage of this approach is that it does not distinguish a 2′-*O*-methyl modification from other potential modifications on the 3′ terminal ribose. In studies by Yu et al., it was possible to assign the identity of the 3′-terminal nucleotide modification thanks to mass spectrometry studies [58]. 

More recently, an alternative chemical treatment which should allow the distinction between unmethylated and 2′-*O*-methylated residues has been proposed [61, 62]. Free unconstrained ribose 2′-OH easily reacts with N-methylisatoic anhydride (NMIA, Figure 4(c)), and the resulting adduct can be analyzed by primer extension (selective 2′-hydroxyl acylation analyzed by primer extension, SHAPE). For the moment, the method was only applied to RNA structural analysis. However, comparisons of NMIA modification and primer extension profiles for unmodified transcript and 2′-*O*-methyl-containing RNA may probably be used as a tool for 2′-*O*-methylation mapping.

Yet another approach allowing the separation of O-methyl nucleosides from ribose-unsubstituted nucleosides in one chromatographic step has been developed in the early 80s [63]. This methods is based on the capability of boronate to form an anionic complex with the cis-2',3' hydroxyls of unsubstituted ribonucleosides, but not with ribose-substituted nucleosides such as 2′-*O*-methylnucleosides. Phenyl boronates with hydrophobic side chains of about 1 nm in length have been synthesized and used to coat inert 10 *μ*m beads of polychlorotrifluoroethylene. This matrix complexes easily with compounds containing free vicinal cis-hydroxyls allowing their separation from their O-alkyl or O-acyl derivatives. Boronates also find application in affinity electrophoresis [64], as will be detailed later on.

### 3.5. Pseudouridine

**Figure 5:**
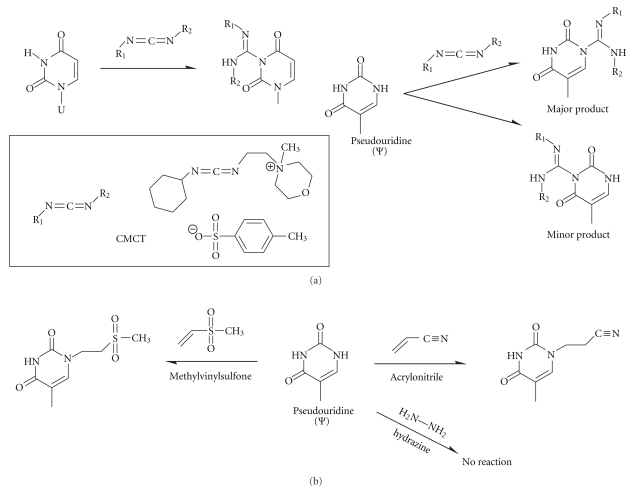
Pseudouridine (Ψ-) specific reagents. (a) Pseudouridine and uridine reactivity with CMCT and similar water-soluble carbodiimides. Structural formula of CMCT is shown on the left. (b) Pseudouridine reactions with methylvinylsulfone and acrylonitrile. Pseudouridine residues are resistant to hydrazine, while unmodified uridines are specifically cleaved (see Figure 2(a)).

Pseudouridine (psi, Ψ) is the result of an isomerization reaction during which the *C5* and N1 positions of uracil are interconverted. The resulting nucleotide features a glycosidic carbon-carbon bond and an additional amide functionality, which is available for chemical discrimination (Figure 5). Because Ψ retains the base pairing characteristics of uridine, it is invisible in RT profiles and must be chemically modified (Figure 5(a)) for detection by either mass spectroscopy [65–67] or by RT [8]. Somewhat surprisingly, chemical modification can be achieved with satisfying selectivity by two classes of electrophiles. Michael acceptors such as acrylonitrile [68] or methylvinylsulfone [69] alkylate the *N3* while not reacting with the four major nucleotides (Figure 5(b)). These reagents are, however, known to react also with inosine, m^5^C, and m^5^U [69, 70]. 

Ψ residues can be acylated on both nitrogens by carbodiimides (Figure 5(a)) [71, 72]. The reaction also acylates both the *N*3 of uridines and the *N1* of guanosines, but all residues except for the one on *N1 *of Ψ can be removed by subsequent alkaline treatment [73, 74]. Indeed, whereas U and G adducts of CMC are readily cleaved by weakly alkaline conditions, cleavage of N3-CMC-Ψ requires 7 M NH_4_OH at 100°C for 8 min [73]. The remaining bulky CMC residues are then detected by primer extension using reverse transcriptase.

Recent extensive studies on pseudouridine mapping by CMCT treatment and MALDI-TOF MS pointed out that modified nucleotide ms^2^i^6^A present in *E. coli* tRNA also retains a CMCT derivative after acylation [67]. However, the chemical basis for this particular reactivity was not elucidated in detail.

Another approach used to detect Ψ residues depends upon the greater resistance of Ψ and m^5^U residues to hydrazinolysis compared to U and most other U-derived bases (Figure 5(c)). The reaction with hydrazine leads to pyrimidine ring opening (see Figure 2(a)). Subsequent aniline treatment cleaves the polynucleotide chain leading to the termination of RT [75–77]. Ψ residues, being resistant to hydrazinolysis, are not cleaved and do not stop RT.

### 3.6. Thiolated Nucleotides

**Figure 6:**
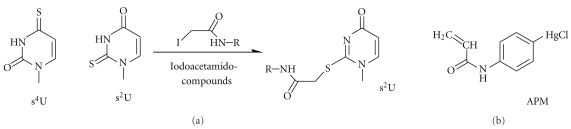
Examples of thiolated nucleotides found in RNA (a) and (*N*-acryloylamino)phenyl-4-mercuric chloride (APM) which is used for gel-retardation detection of thiolated molecules. (b) Reaction of thiolated nucleotides with iodoacetamide and its derivatives. s^2^U is taken to illustrate the reaction product.

Sulfur atoms, even when introduced into RNA as thiocarbonyl functions, are easily oxidized but are also very nucleophilic and thus react with a variety of electrophiles under conditions under which the remainder of RNA residues stays inert. Iodoacetamide derivatives, for example, of fluorescent dyes, are typical reagents to derivatize sulfur residues in proteins, that is, free cysteins. Diluted solutions of these reagents have been shown to selectively conjugate to thiolated nucleotides such as s^2^U, s^4^U, and s^2^C [43, 44] (Figure 6). Thiouridines have also been reported to react with carbodiimides like CMCT [78], and the resulting conjugates can be identified by mass spectroscopy [65, 67].

The thiophilic character of mercury ions has been exploited for affinity electrophoresis in polyacrylamide gels copolymerized with (*N*-acryloylamino)phenyl-4-mercuric chloride (APM-gels, Figure 6). Sulfur-containing RNAs are retarded during migration, and interestingly, the degree of retardation depends on the nature of the thiomodification; s^4^U is more strongly retarded comparing to s^2^U [79].

### 3.7. Modifications Containing Primary Amines

**Figure 7:**
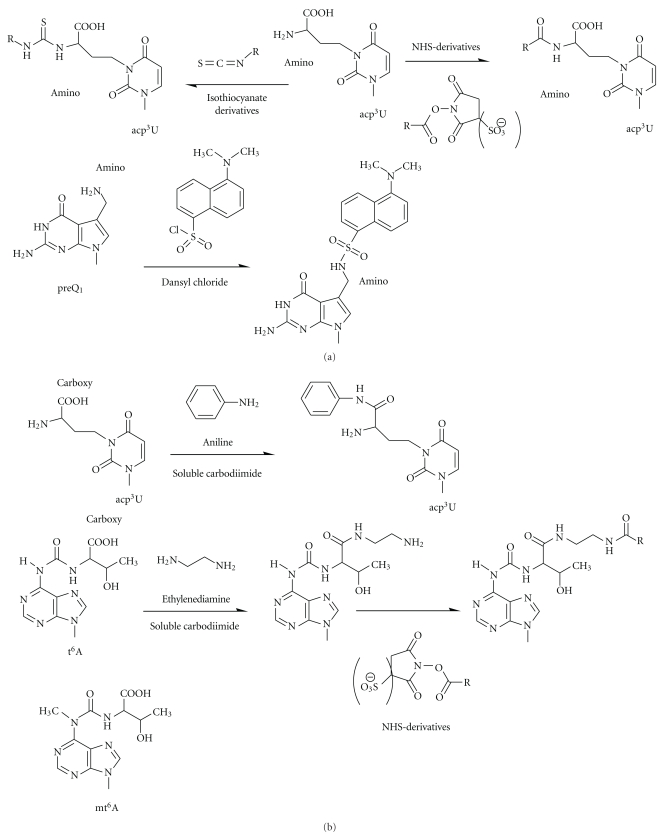
Specific reactivity of free NH_2_– (a) and –COOH (b) groups in modified nucleotides. acp^3^U is taken to illustrate the reactivity with isothiocyanate derivatives and NHS derivatives. Reaction with dansyl chloride is shown for preQ_1_ (a). Reactions of free –COOH groups with aniline, ethylenediamine and similar molecules in the presence of soluble carbodiimide. The resulting free NH_2_-group of ethylenediamine may be further used for attachment of activated acyl (b). The structure of mt^6^A(m^6^t^6^A) is shown at the bottom.

Primary amines, which are not present in unmodified RNA, are relatively strong nucleophiles which can be selectively conjugated to a number of commonplace amino-derivatizing agents containing, for example,* N*-hydroxysuccinimide and isothiocyanate moieties [78, 80, 81], and probably with pentafluorophenol derivatives, too (Figure 7(a)). For example, labeling with fluorescent dyes has been successfully implemented for *E. coli* tRNA^Phe^ containing acp^3^U [78, 81]. Fluoresceinisothiocyanate under mild alkaline conditions was successfully applied for the labeling of *E. coli* tRNA^Tyr^ at the queosine position [82]. Free amino functions may also react with dansyl chloride under neutral conditions, as was demonstrated by selective labeling of preQ_1_-34 in the same *E. coli* tRNA^Tyr^ obtained from queosine-deficient strain [83] (Figure 7(a)).

### 3.8. Modifications Containing free Carboxylic Acid Groups

A number of modifications typically occurring at positions 20, 34, and 37 of tRNAs consist in the incorporation of an amino acid via its amino function, resulting in the presence in the modified RNA of a free carboxy group. This can be selectively activated for conjugation with amine reagents by dilute water-soluble carbodiimide, which will not react with the other nucleobases under these conditions (Figure 7(b)). The principle has been demonstrated with mt^6^A (m^6^t^6^A) and acp^3^U [45, 46] but may be applicable to a variety of other modifications, potentially including ms^2^t^6^A, m^6^t^6^A, g^6^A, hn^6^A, and ms^2^hn^6^A, chm^5^U, cmo^5^U, cmnm^5^s^2^U, cmnm^5^Um, cmnm^5^U, m^1^acp^3^Ψ, *τ*m^5^U, and *τ*m^5^s^2^U and possibly others.

### 3.9. Dihydrouridine

**Figure 8:**
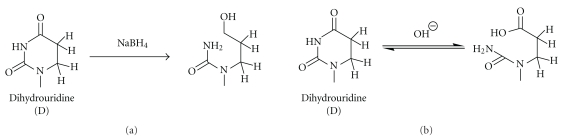
Cleavage of the dihydrouridine ring (a) upon reduction by sodium borohydride (NaBH_4_) and (b) at mild alkaline conditions. In both cases, cleavage of the dihydrouridine ring is followed by cleavage of the RNA chain.

In contrast to all other pyrimidines, the dihydrouridine (D) has a saturated pyrimidine ring due to reduction of the double C=C bond between carbons 5 and 6. This considerably affects the stability of D both in the reduction by NaBH_4_ [84–86] and under alkaline conditions [87]. Initial studies of tRNA nucleosides by NaBH_4_ treatment revealed that D, ac^4^C, and s^4^U are reduced under rather harsh reaction conditions [84], while major nucleosides, as well as Ψ residues, were found to be inert. The product of D reduction by NaBH_4_ is a ureidopropanol riboside (Figure 8(a)). Further studies demonstrated that the D residue in tRNA can be selectively reduced by NaBH_4_ under very mild conditions at pH 7.5 in Tris-HCl buffer on ice [86].

Another characteristic of D is its particular sensitivity to alkaline hydrolysis. Hydrolytic ring opening (Figure 8(b)) of dihydrouridine occurs quite rapidly at elevated temperatures and a pH above 8.0–8.5 [88]. This instability may explain the absence of this modification in hyperthermophiles and was also used for detection of D residues in yeast tRNA and characterization of the corresponding tRNA D-syntases [87].

### 3.10. Inosine

**Figure 9:**
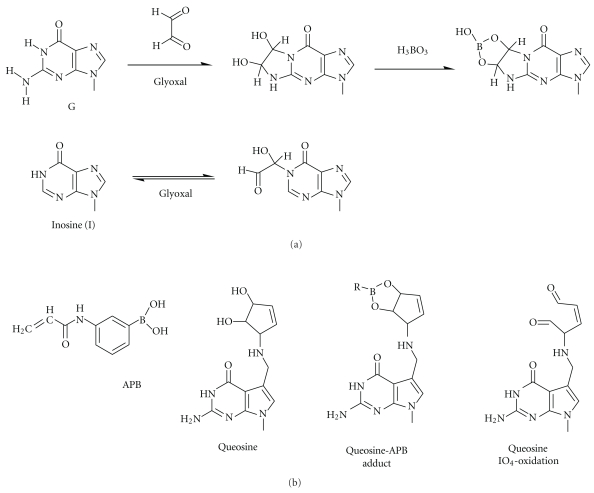
Reaction of guanosine residues in RNA with glyoxal and stabilization of the resulting adduct by boric acid H_3_BO_3_. Inosine residues do not form the stable product under similar conditions (a). Organic derivative of boric acid (*N*-acryloylamino)phenyl-3-boronic acid (APB) and its complex with cisdiols present in queosine and the dialdehyde resulting from periodate oxydation of the cisdiol (b).

The modified nucleotide inosine (I) is derived from adenosine (A) by enzymatic deamination catalyzed by specific adenosine deaminases [89, 90]. RNase T_1_ does not distinguish inosine residues present in RNA from guanosine residues and cleaves on their 3′ side. However, preliminary specific reaction with glyoxal allows RNase T_1_ to distinguish G and I residues [91] (Figure 9(a)). Indeed, glyoxal reacts with *N1* and *N^2^* of G residues and thus abolishes their recognition by RNase T_1_. The resulting covalent adduct is stabilized by boric acid. By contrast, inosine cannot react in the same way with glyoxal due to the absence of NH_2_-group at position 2 and the glyoxal-inosine adduct is unstable. Thus, RNase T_1_ cleavage of glyoxal-treated RNA proceeds only at inosine residues and not at G residues. The cleavage positions may be detected by primer extension analysis or other methods.

### 3.11. Queosine

The complicated hypermodification queuosine contains two vicinal hydroxyl groups, a feature otherwise only present in the most 3′-ribose of an RNA chain. A chelating effect of these hydroxyl groups can be exploited by complexation to Lewis acids such as boronic acid. Igloi and Kössel [64] have exploited this feature to develop affinity electrophoresis based on the incorporation of (*N*-acryloylamino)phenyl-3-boronic acid (APB) into polyacrylamide gels (Figure 9(b)). Affinity chromatography based on the same principle has been described by Vogeli et al. [92]. Of note, vicinal hydroxyl groups can be selectively oxidatized to yield dialdehydes which can be selectively condensed with hydrazine derivatives, yielding hydrazones that can be further stabilized by reduction with borohydrides.

### 3.12. Wybutosine

**Figure 10:**
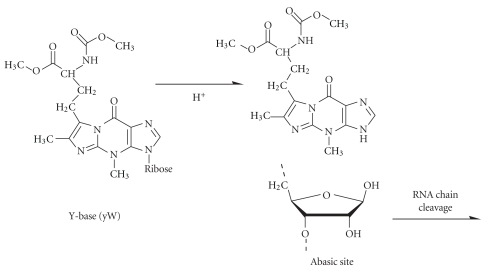
Depurination of wybutosine base (yW, Y-base) under mild acidic conditions. The formation of abasic site in RNA leads to the subsequent cleavage of the RNA chain.

The reaction of the wybutosine base (yW) in yeast tRNA^Phe^ with NaBH_4_ has already been mentioned. Its depurination under mild HCl treatment (pH 2.9, 37°C, 2-3 hours, Figure 10) was already noticed in the late 60s and 70s [17, 93]. Later on, the reaction of the riboaldehyde group formed at the resulting abasic site with hydrazine and hydrazine derivatives was used for specific fluorescence labeling of tRNA^Phe^ for biophysical studies of the ribosomal translation [86, 94].

## 4. Practical Use of Specific Reagents for Detection of Modified Nucleotides by RT or RNA Chain Cleavage

Specific chemicals like those described above may be used both for detection and the precise localization of modified residues in RNAs. The techniques developed for RNA sequencing using Maxam and Gilbert's approach also allow for detection of modified nucleotides, since many chemical reactions that are specific for modified nucleotides may be exploited to induce cleavage of RNA polynucleotide chain, often in combination with further chemical treatment. This is the case, for instance, for hydrazine cleavage, NaBH_4_ reduction, or hydrolysis of phosphodiester bonds under various conditions. Analysis of the cleavage position can be performed with 5′-^32^P-labeled RNA and separation of the cleaved fragments by electrophoresis on denaturing PAGE. However, these techniques require difficult and time-consuming purification to homogeneity of a sufficient amount (at least 0.5–1 *μ*g) of a given RNA species and thus cannot be applied to low-abundance cellular RNAs.

An alternative approach for the detection of RNA modifications is based on the use of RT. A synthetic DNA oligonucleotide designed to target a specific sequence is annealed to cellular RNA without previous RNA purification. Primer elongation by reverse transcriptase allows both sequencing of RNA species by Sanger type reactions and, in addition, the detection of specific reagent-dependent stops, which are indicative of the presence and exact position of modified nucleotides. A limited number of natural RNA modifications altering Watson-Crick base pairing can be detected by this approach even in the absence of a preceding chemical treatment. For RT-silent modifications, a specific reagent is required to create a bulky chemical modification, which blocks base pairing and consequently progression of the reverse transcriptase, thus resulting in an RT stop. Alternatively, cleavages of the RNA chain generated by specific treatment may also be detected by primer extension. However, interpretation of RT profiles should be undertaken with caution, since the presence of stops or gaps in the RT profile is frequently hidden by natural pauses of the RT at the corresponding positions. 

For some RNA modifications, a simple primer extension under specific conditions may also be used for detection. Such method was developed for the precise mapping of 2′-*O*-methylated residues in RNA and uses primer extension by reverse transcriptase at low dNTP concentrations [55, 56]. It is based on the observation that limited dNTP concentrations cause the reverse transcriptase to frequently slow down (or pause) at such nucleotides. Primer extension with normal (unmodified) residues is much less sensitive to reduced dNTP concentration. Many other modified nucleotides in RNA do not create such pauses, and thus low dNTP concentration-dependent stops seem to be rather specific to 2′-*O*-methylation. The comparison of RT profile for natural (modified) RNA and unmodified RNA transcript may help to discriminate modification-dependent from other RT stops. It is noteworthy that pauses observed at 2′-*O*-methylated residues are sequence dependent and, depending on the sequence context, no pause may occur at some of the 2′-*O*-methylated residues. Therefore, the method does not allow an exhaustive identification of all 2′-*O*-methylated residues in RNA molecules. In some instances, RNA sequence changes resulting from RNA editing (e.g., adenosine or cytidine deamination) can also be directly detected by comparison of RT profile for unmodified transcript and natural RNA species.

Other methods employing RT are based on particular base pairing properties of some modified nucleotides. An alternative approach for inosine detection consists in direct RNA sequencing with RT and comparison of the cDNA sequence obtained with the corresponding genomic sequence. Due to the absence of amino group at position 6, I base pairs with C residues instead of U residues, thus change the sequence of the cDNA synthesized by RT. Thus, one can detect unexpected A → G changes in the cDNA compared to the genomic sequence [95]. To confirm the data, it is also possible to compare the cDNA sequence obtained by direct sequencing of an unmodified transcript produced *by in vitro* transcription with the cDNA sequence obtained with the authentic RNA. Another recently developed method that is suitable for transcriptome-wide analysis of inosine is based on specific cyanoethylation of inosine [70].

## 5. Conclusion

Despite extensive development of specific chemical reagents during several decades (starting in the 1970s), the spectrum of available chemicals capable of selective reaction with modified ribonucleotides remains rather limited. This is related to a limited difference of chemical reactivity between modified nucleotides and their unmodified counterparts. Furthermore, the low stability of RNA molecules during required reaction conditions presents a stringent limitation. Due to these limitations, development of specific reaction schemes was possible only for a small subset of the known modified residues in RNA. Future development in the field should fill these gaps and propose new chemical reagents for extensive analysis of RNA modification by RT and by MS techniques.

## Figures and Tables

**Table 1:** Known RNA modifications and their abbreviations and symbols (modified nucleosides mentioned in the text are indicated in bold).

Number	Symbol	Common name
(1)	**m^1^A **	**1-methyladenosine **
(2)	m^2^A	2-methyladenosine
(3)	m^6^A	*N^6^*-methyladenosine
(4)	**Am **	2′**-*O*-methyladenosine **
(5)	ms^2^m^6^A	2-methylthio-*N^6^*-methyladenosine
(6)	i^6^A	*N^6^*-isopentenyladenosine
(7)	**ms^2^i^6^A **	**2-methylthio-** *N^6^* **-isopentenyladenosine **
(8)	io^6^A	*N^6^*-(*cis*-hydroxyisopentenyl)adenosine
(9)	ms^2^io^6^A	2-methylthio-*N^6^*-(*cis*-hydroxyisopentenyl) adenosine
(10)	**g^6^A **	***N*** ^6^ **-glycinylcarbamoyladenosine **
(11)	**t^6^A **	***N*** ^6^ **-threonylcarbamoyladenosine **
(12)	**ms^2^t^6^A **	**2-methylthio-*N *** ^6^ **-threonyl carbamoyladenosine **
(13)	**m^6^t^6^A **	***N*** ^6^ **-methyl-*N *** ^6^ **-threonylcarbamoyladenosine **
(14)	**h** **n** ^6^ **A **	***N*** ^6^ **-hydroxynorvalylcarbamoyladenosine **
(15)	**ms^2^h** **n** ^6^ **A **	**2-methylthio-*N *** ^6^ **-hydroxynorvalyl carbamoyladenosine **
(16)	Ar(p)	2′-*O*-ribosyladenosine (phosphate)
(17)	**I**	**inosine **
(18)	m^1^I	1-methylinosine
(19)	m^1^Im	1,2′-*O*-dimethylinosine
(20)	**m^3^C **	**3-methylcytidine **
(21)	**m^5^C **	**5-methylcytidine **
(22)	**Cm **	**2** ^**'**^ **-*O*-methylcytidine **
(23)	**s^2^C **	**2-thiocytidine **
(24)	**ac^4^C **	***N*** ^4^ **-acetylcytidine **
(25)	f^5^C	5-formylcytidine
(26)	m^5^Cm	5,2′-*O*-dimethylcytidine
(27)	ac^4^Cm	***N*** ^4^-acetyl-2′-*O*-methylcytidine
(28)	**k^2^C **	**lysidine **
(29)	m^1^G	1-methylguanosine
(30)	m^2^G	*N^2^*-methylguanosine
(31)	**m^7^G **	**7-methylguanosine **
(32)	**Gm **	**2** ^**'**^ **-*O*-methylguanosine **
(33)	m^2^_2_G	*N^2^*,*N^2^*-dimethylguanosine
(34)	m^2^Gm	*N^2^*,2′-*O*-dimethylguanosine
(35)	m^2^_2_Gm	*N^2^*,*N^2^*,2′-*O*-trimethylguanosine
(36)	Gr(p)	2′-*O*-ribosylguanosine (phosphate)
(37)	**yW **	**wybutosine **
(38)	o_2_yW	peroxywybutosine
(39)	OHyW	hydroxywybutosine
(40)	OHyW*	undermodified hydroxywybutosine
(41)	imG	wyosine
(42)	mimG	methylwyosine
(43)	**Q**	**queuosine **
(44)	oQ	epoxyqueuosine
(45)	galQ	galactosyl-queuosine
(46)	manQ	mannosyl-queuosine
(47)	preQ_0_	7-cyano-7-deazaguanosine
(48)	**preQ_1_**	**7-aminomethyl-7-deazaguanosine **
(49)	G^+^	archaeosine
(50)	**Ψ**	**pseudouridine **
(51)	**D**	**dihydrouridine **
(52)	**m^5^U **	**5-methyluridine **
(53)	**Um **	**2** ^**'**^ **-*O*-methyluridine **
(54)	m^5^Um	5,2′-*O*-dimethyluridine
(55)	m^1^Ψ	1-methylpseudouridine
(56)	Ψm	2′-*O*-methylpseudouridine
(57)	**s^2^U **	**2-thiouridine **
(58)	**s^4^U **	**4-thiouridine **
(59)	m^5^s^2^U	5-methyl-2-thiouridine
(60)	s^2^Um	2-thio-2′-*O*-methyluridine
(61)	**acp^3^U **	**3-(3-amino-3-carboxypropyl)uridine **
(62)	ho^5^U	5-hydroxyuridine
(63)	mo^5^U	5-methoxyuridine
(64)	cmo^5^U	uridine 5-oxyacetic acid
(65)	mcmo^5^U	uridine 5-oxyacetic acid methyl ester
(66)	**chm^5^U **	**5-(carboxyhydroxymethyl)uridine **
(67)	mchm^5^U	5-(carboxyhydroxymethyl)uridine methyl ester
(68)	mcm^5^U	5-methoxycarbonylmethyluridine
(69)	mcm^5^Um	5-methoxycarbonylmethyl-2′-*O*-methyluridine
(70)	mcm^5^s^2^U	5-methoxycarbonylmethyl-2-thiouridine
(71)	nm^5^s^2^U	5-aminomethyl-2-thiouridine
(72)	mnm^5^U	5-methylaminomethyluridine
(73)	mnm^5^s^2^U	5-methylaminomethyl-2-thiouridine
(74)	mnm^5^se^2^U	5-methylaminomethyl-2-selenouridine
(75)	ncm^5^U	5-carbamoylmethyluridine
(76)	ncm^5^Um	5-carbamoylmethyl-2′-*O*-methyluridine
(77)	**cmnm^5^U **	**5-carboxymethylaminomethyluridine **
(78)	**cmnm^5^Um **	**5-carboxymethylaminomethyl-2** ^**'**^ **-*O*-methyluridine **
(79)	**cmnm^5^s^2^U **	**5-carboxymethylaminomethyl-2-thiouridine **
(80)	m^6^_2_A	*N^6^*,*N^6^*-dimethyladenosine
(81)	Im	2′-*O*-methylinosine
(82)	m^4^C	*N^4^*-methylcytidine
(83)	m^4^Cm	*N^4^*,2′-*O*-dimethylcytidine
(84)	hm^5^C	5-hydroxymethylcytidine
(85)	m^3^U	3-methyluridine
(86)	**m^1^acp^3^Ψ **	**1-methyl-3-(3-amino-3-carboxypropyl) pseudouridine **
(87)	cm^5^U	5-carboxymethyluridine
(88)	m^6^Am	*N^6^*,2′-*O*-dimethyladenosine
(89)	m^6^_2_Am	*N^6^*,*N^6^*,2′-*O*-trimethyladenosine
(90)	m^2,7^G	*N^2^*,7-dimethylguanosine
(91)	m^2,2,7^G	*N^2^*,*N^2^*,7-trimethylguanosine
(92)	m^3^Um	3,2′-*O*-dimethyluridine
(93)	m^5^D	5-methyldihydrouridine
(94)	m^3^Ψ	3-methylpseudouridine
(95)	f^5^Cm	5-formyl-2′-*O*-methylcytidine
(96)	m^1^Gm	1,2′-*O*-dimethylguanosine
(97)	m^1^Am	1,2′-*O*-dimethyladenosine
(98)	***τ*m^5^U **	**5-taurinomethyluridine **
(99)	***τ*m^5^s^2^U **	**5-taurinomethyl-2-thiouridine **
(100)	imG-14	4-demethylwyosine
(101)	imG2	isowyosine
(102)	ac^6^A	*N^6^*-acetyladenosine
(103)	inm^5^U	5-(isopentenylaminomethyl)uridine
(104)	inm^5^s^2^U	5-(isopentenylaminomethyl)-2-thiouridine
(105)	inm^5^Um	5-(isopentenylaminomethyl)-2′-*O*-methyluridine
(106)	m^2,7^Gm	*N^2^*,7,2′-*O*-trimethylguanosine
(107)	m^4^_2_Cm	*N^4^*, *N^4^*,**2** ^**'**^-*O*-trimethylcytidine
(108)	m^8^A	8-methyladenosine

Numbering and abbreviations of modified nucleosides are from “The RNA modification Database,” http://s59.cas.albany.edu/RNAmods/.

## Abbreviations

APM: (*N*-acryloylamino)phenyl-4-mercuric chloride
APB: (*N*-acryloylamino)phenyl -3-boronic acid
RT: Reverse transcription
TLC: Thin layer chromatography
CMCT: 1-cyclohexyl-3-(2-(4-morpholinyl)ethyl)*carbodiimide* tosylate
DMS: Dimethylsulfate
DEPC: Diethyl pyrocarbonate
DMAS-Cl: N,N-(dimethylamino)dimethylchlorosilane
NHS: N-hydroxysuccinimide.
